# Metastatic Lung Adenocarcinoma Presenting With Cavitary and Consolidative Lung Findings: A Diagnostic Dilemma

**DOI:** 10.1155/crom/5007092

**Published:** 2025-07-17

**Authors:** Abdalhakim Shubietah, Omar Hamadi, Mahmoud Doudein, Malak Ramzy Hroub, Muath Baniowda, Amer Abu-Shanab, Khalil Karim, Suliman Hamadneh

**Affiliations:** ^1^Department of Internal Medicine, Advocate Illinois Masonic Medical Center, Chicago, Illinois, USA; ^2^Department of Internal Medicine, Al-Istishari Arab Hospital, Ramallah, West Bank, State of Palestine; ^3^Faculty of Medicine, Al-Quds University, Jerusalem, West Bank, State of Palestine; ^4^Department of Internal Medicine, University of Missouri-Kansas City, Kansas City, Missouri, USA; ^5^Department of Internal Medicine, Rutgers-Monmouth Medical Center, Long Branch, New Jersey, USA; ^6^Pulmonary Consultant, Head of Pulmonary and Bronchoscopy Unit, Al-Istishari Arab Hospital, Ramallah, West Bank, State of Palestine; ^7^Internal Medicine Specialist, Al-Istishari Arab Hospital, Ramallah, West Bank, State of Palestine

**Keywords:** cavitary lung lesions, metastatic lung adenocarcinoma, non–small cell lung cancer

## Abstract

**Introduction:** Lung cancer is the leading cause of cancer-related mortality, with non–small cell lung cancer (NSCLC) accounting for 85% of cases. Lung adenocarcinoma, the most common subtype, can mimic benign conditions like pneumonia, lung abscess, and interstitial lung disease due to its varied radiologic presentations and associated inflammation and fibrosis. This similarity can delay diagnosis, emphasizing the need for imaging and histopathological confirmation.

**Case Presentation:** A 58-year-old male with a significant smoking history, hypertension, and GERD presented with a 5-month history of episodic epigastric pain, exacerbated by heavy meals, along with progressive respiratory symptoms, including shortness of breath, dry cough, and a 20-kg weight loss over 3 months. Upper endoscopy revealed gastric ulcers, a hiatal hernia, esophageal mucosal changes consistent with GERD, and *Helicobacter pylori* infection on biopsy, which was treated with triple therapy, resolving his gastrointestinal symptoms. However, his respiratory symptoms worsened, with increased dyspnea at rest, pleuritic chest pain, and a persistent cough. Chest CT showed multiple cavitating lung nodules, architectural distortion predominantly in the upper lobes, a large irregular lesion in the right lower lobe, and enlarged paratracheal, subcarinal, and distal paraesophageal lymph nodes. The patient was transferred to our facility for further evaluation. Whole-body CT revealed widespread bilateral cavitary lesions, lymphadenopathy, and a small hiatal hernia. Bronchoscopy with biopsy confirmed metastatic lung adenocarcinoma, with histopathology showing moderately differentiated adenocarcinoma, positive for TTF-1 and Napsin A and negative for PAX8. Cytology from bronchoalveolar lavage also confirmed malignancy, and PD-L1 immunostaining showed weak positivity in 15%–20% of tumor cells. The patient was diagnosed with metastatic lung adenocarcinoma and initiated on carboplatin and pemetrexed chemotherapy. Molecular testing was planned, and he was discharged for follow-up care.

**Conclusion:** Our case of a 58-year-old male with cavitating lung nodules, significant weight loss, and progressive respiratory symptoms, initially misattributed to gastrointestinal disease, highlights the diagnostic complexity of lung adenocarcinoma. The biopsy-confirmed diagnosis of metastatic adenocarcinoma underscores the need for clinicians to maintain a high index of suspicion for malignancy in patients with atypical or nonspecific presentations. Early tissue diagnosis is crucial for timely treatment and improved outcomes, especially in cases involving cavitary lesions or persistent, unexplained symptoms.

## 1. Introduction

Cavitary pulmonary nodules pose a significant diagnostic challenge, as they can result from a wide range of benign and malignant conditions. According to the Fleischner Society, a cavity is defined as a gas-filled space within pulmonary consolidation, a mass, or a nodule, with specific characteristics such as wall thickness and morphology aiding in differentiating underlying etiologies [1].

Historically, squamous cell carcinoma has been the most common histologic subtype associated with cavitation. However, recent evidence indicates that lung adenocarcinoma can also present with cavitary lesions [2]. Cavitation has been reported in up to 22% of primary lung cancer cases, a phenomenon attributed to rapid tumor growth outpacing its blood supply, leading to central necrosis [3].

While cavitation is relatively common in primary lung cancer, it remains rare in metastatic disease, occurring in only 4% of lung metastases [4]. This makes our case a unique one.

## 2. Case Presentation

A 58-year-old male with a history of hypertension and gastroesophageal reflux disease (GERD) was in his usual state of health until 5 months prior to admission, when he developed episodic epigastric pain, exacerbated by heavy meals, accompanied by shortness of breath and a dry cough, which worsened at night and interfered with sleep. Upper endoscopy revealed multiple gastric ulcers, a hiatal hernia, esophageal mucosal changes consistent with GERD, and *Helicobacter pylori* infection (on biopsy). He was treated with triple therapy for *H. pylori*, followed by pantoprazole and over-the-counter antacids, leading to the resolution of gastrointestinal symptoms. However, his respiratory symptoms progressively worsened, leading to reduced exercise tolerance, persistent dry cough, and a 20-kg weight loss over 3 months.

**Figure 1:**
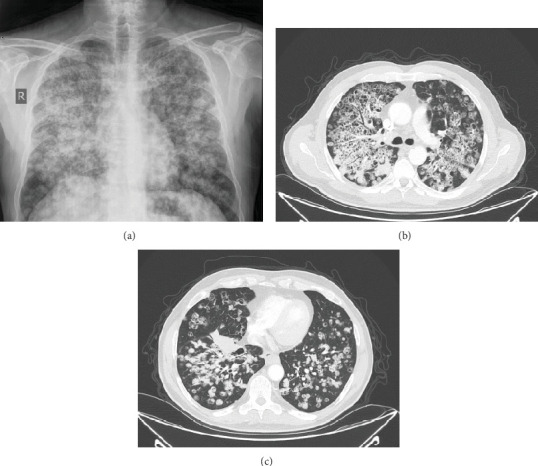
(a) Chest x-ray: diffuse bilateral reticulonodular opacities with upper and midlung consolidations, indicating extensive parenchymal involvement. (b, c) Axial chest CT: multiple cavitary nodules with irregular margins and architectural distortion.

Two days before admission, his respiratory status acutely declined, marked by dyspnea at rest, increased work of breathing, pleuritic chest pain, and a persistent dry cough. A chest radiograph demonstrated diffuse bilateral reticulonodular opacities with upper- and midlung consolidations, consistent with extensive parenchymal involvement. Subsequent chest CT confirmed multiple cavitary nodules of varying sizes, architectural distortion predominantly in the upper lobes, and confluent opacities with air bronchograms in the midlung fields; it also revealed a 5 × 3 × 4 cm irregular lesion in the anterior superior segment of the right lower lobe, a 3 × 2.5 cm deep hilar nodule encasing adjacent vessels, and multiple enlarged paratracheal, subcarinal, and distal paraesophageal lymph nodes (largest 2 × 1.5 cm). He denied fever, chills, night sweats, hemoptysis, orthopnea, peripheral edema, neurological symptoms, rash, hematuria, or immunosuppressant use. His family history was notable for asthma in his father and uncle, and he described episodic nocturnal dyspnea relieved by deep inhalation (Figures 1a, 1b, and 1c).

The patient was transferred on 30/12/2024 for further evaluation of cavitary lung lesions. Differential diagnosis included malignancy, tuberculosis, aspergillosis, and vasculitis. Oncology whole-body CT with IV contrast showed innumerable bilateral cavitating lung nodules causing architectural distortion, more prominent in the upper lobes, with a 5.8 × 3.2 × 2.8 cm irregular mass in the right lower lung lobe just above the diaphragm, an ill-demarcated infiltrative process along the right hilar bronchovascular bundle, mediastinal and hilar lymphadenopathy (largest measuring 3.4 × 2.3 cm), a prominent lymph node in the right paratracheal region of the thoracic inlet measuring approximately 8 mm in short axis, a small hiatal hernia, a few bilateral hypodense thyroid foci, mild thickening of the left adrenal gland, a few left renal cortical cysts (largest 4 cm), and liver hypodensities suggestive of focal fatty infiltration. Brain, bone, and other abdominal organs were unremarkable.

Bronchoalveolar lavage (BAL) fluid from the right middle lobe was submitted for bacterial culture, acid-fast bacilli (AFB) smear and culture, and cytological analysis. All cultures—including routine bacterial and mycobacterial—remained sterile, and AFB staining was negative. Cytological analysis of the BAL fluid did not reveal malignant cells. Additionally, peripheral blood and sputum cultures yielded no growth.

Bronchoscopy with endobronchial ultrasound-guided transbronchial biopsy confirmed the diagnosis of metastatic lung adenocarcinoma. Histopathological evaluation revealed a moderately differentiated, invasive adenocarcinoma with neoplastic cells positive for thyroid transcription factor-1 (TTF-1) and Napsin A and negative for paired box gene 8 (PAX8).

Programmed death-ligand 1 (PD-L1) immunohistochemistry demonstrated weak membranous staining in 15%–20% of tumor cells. HER2/neu immunostaining was negative (score 0).

The diagnostic tissue was obtained via C-arm–guided transbronchial biopsies from lesions in the right upper and middle lobes. Specimens were submitted for both histopathological assessment and tissue culture. Microscopic examination revealed a moderately differentiated invasive adenocarcinoma with extensive central tumor necrosis and fibrin deposition; no hemorrhage was observed.

Comprehensive molecular profiling identified an oncogenic *ERBB2* p.Gly776delinsValCys mutation within the kinase domain, known to enhance protein activation and promote cellular proliferation. This mutation conferred resistance to trastuzumab, lapatinib, and erlotinib, yet retained sensitivity to afatinib, neratinib, and osimertinib. Although these agents are not immunotherapeutics, it is noteworthy that neither they nor immune checkpoint inhibitors such as pembrolizumab, nivolumab, or atezolizumab were available at our institution or within the country at the time treatment was initiated.

Carboplatin plus pemetrexed chemotherapy was initiated, and Oncomine next-generation sequencing was ordered to guide subsequent therapy. The patient was discharged on 11 January 2025. Six weeks after starting treatment (Postcycle 2), chest CT showed an approximate 25% reduction in tumor burden; at 12 weeks (completion of four cycles), restaging CT demonstrated stable disease with no additional change.

## 3. Discussion

The diagnostic challenge in this case arose from the patient's progressive respiratory symptoms, significant weight loss, and gastrointestinal complaints. Despite treatment for *H. pylori* and GERD, his condition worsened. Initial chest radiography demonstrated diffuse bilateral reticulonodular opacities with upper- and midlung consolidations, indicating extensive parenchymal involvement. CT imaging revealed cavitating lung nodules, architectural distortion, and lymphadenopathy. The differential diagnosis included infection, interstitial lung disease, granulomatous disease, and malignancy. Biopsy confirmed metastatic lung adenocarcinoma, with histopathology showing moderately differentiated tumor cells positive for TTF-1 and Napsin A and weak PD-L1 expression in 15%–20% of cells.

Similar cases in the literature underscore this diagnostic complexity. Cengiz et al. described a 74-year-old male nonsmoker whose interstitial lung findings were ultimately diagnosed as minimally invasive adenocarcinoma [5]. Mehta et al. reported a 59-year-old woman initially treated for ILD whose biopsy later revealed bronchoalveolar carcinoma [6]. These cases emphasize the importance of maintaining a high index of suspicion for malignancy, particularly in patients with atypical presentations or inadequate responses to empirical therapies. In a case by Carreto et al., a 62-year-old former smoker presented with chronic dry cough, fatigue, and weight loss over 5 months; imaging revealed multiple bilateral thin-walled cavities and centrilobular nodules predominantly in basal regions. Bronchoscopy was normal, and BAL results were negative, although autoimmune screening showed positive ANA and anti-SSA antibodies. Surgical lung biopsy confirmed mucinous adenocarcinoma, leading to a diagnosis of Stage IV lung cancer, subsequently managed with chemotherapy [7].

Cavitary lung cancers arise through multiple overlapping mechanisms. The most frequent is ischemic necrosis or secondary abscess formation from tumor overgrowth of feeding vessels, combined with bronchiolar obstruction [8]. Other contributors include neoplastic transformation of the cavity wall, protease- or mucin-mediated destruction of the alveolar septa, and a bronchiolar “check-valve” effect, where tumor infiltration narrows terminal bronchioles, permitting air entry on inspiration but impeding its exit on expiration, leading to progressive air trapping and cystic degeneration of the mass [8–10]. These processes collectively confer the high malignant potential observed in cavitary lung cancers [9, 11].

## 4. Conclusion

Our case highlights the critical need for clinicians to maintain vigilance for malignancy in patients presenting with cavitary and consolidative pulmonary lesions, particularly when accompanied by significant weight loss and inadequate response to empirical therapies. Although historically associated predominantly with squamous cell carcinoma, emerging evidence increasingly recognizes cavitation as a radiologic feature in lung adenocarcinoma, reflecting aggressive tumor biology.

## Data Availability

Data sharing is not applicable to this article as no datasets were generated or analyzed during the current study.
